# Recognizing Ion Ligand–Binding Residues by Random Forest Algorithm Based on Optimized Dihedral Angle

**DOI:** 10.3389/fbioe.2020.00493

**Published:** 2020-06-12

**Authors:** Liu Liu, Xiuzhen Hu, Zhenxing Feng, Shan Wang, Kai Sun, Shuang Xu

**Affiliations:** College of Sciences, Inner Mongolia University of Technology, Hohhot, China

**Keywords:** binding residues, dihedral angle, information entropy, ion ligands, protein

## Abstract

The prediction of ion ligand–binding residues in protein sequences is a challenging work that contributes to understand the specific functions of proteins in life processes. In this article, we selected binding residues of 14 ion ligands as research objects, including four acid radical ion ligands and 10 metal ion ligands. Based on the amino acid sequence information, we selected the composition and position conservation information of amino acids, the predicted structural information, and physicochemical properties of amino acids as basic feature parameters. We then performed a statistical analysis and reclassification for dihedral angle and proposed new methods on the extraction of feature parameters. The methods mainly included applying information entropy on the extraction of polarization charge and hydrophilic–hydrophobic information of amino acids and using position weight matrices on the extraction of position conservation information. In the prediction model, we used the random forest algorithm and obtained better prediction results than previous works. With the independent test, the Matthew's correlation coefficient and accuracy of 10 metal ion ligand–binding residues were larger than 0.07 and 52%, respectively; the corresponding evaluation values of four acid radical ion ligand–binding residues were larger than 0.15 and 86%, respectively. Further, we classified and combined the phi and psi angles and optimized prediction model for each ion ligand–binding residue.

## Introduction

The protein is the foundation of life and plays an important role in the life activities. Proteins carried out various functions by interactions, such as protein–protein interaction, protein–DNA interaction, protein–RNA interaction, and protein–ion ligand interaction (Lin et al., 2005; Ebert and Altman, 2008; Pan and Shen, 2018; Al-Mugotir et al., 2019; Emamjomeh et al., 2019; Robin et al., 2019). Because more than half of the proteins required binding with ion ligands for functions, research of ion ligand–binding residues on proteins was of great significance. However, it was difficult to accurately predict the ion ligand–binding residues on the protein sequence because of the small size and high versatility of ion ligands.

Current theoretical prediction methods of ligand-binding residues can be roughly classified into sequence-based method and three-dimensional (3D) structure–based method. The experiments showed that the accuracy of the 3D structure–based prediction was higher than that of sequence-based prediction (Yang et al., 2013, 2018). However, the number of proteins with known amino acid sequence was far more than that with known 3D structure. Although the prediction accuracy of sequence-based method is not as satisfactory as 3D structure–based, sequence-based method is still generally used.

In general, there were three key points in predicting the ion ligand–binding residues with theoretical methods: research objects selection, the selection, and extraction of feature parameters, and the selection of algorithms. In recent decades, researchers conducted different studies on binding residues of metal and acid radical ion ligands. In these studies, most researchers were working on the development of features to improve the prediction results of ion ligand–binding residues. Among the feature parameters used to predict ion ligand–binding residues, position conservation information, and composition of amino acids were two commonly used basic feature parameters (Sodhi et al., 2004; Komiyama et al., 2015; Hu et al., 2016a; Liu et al., 2019; Wang et al., 2019). Besides, based on the biological background of interaction between ion ligands and proteins, researchers added physicochemical properties of amino acids, secondary structure, and relative solvent accessibility (RSA) to identify ion ligand–binding residues (Lin et al., 2006; Jiang et al., 2016; Cao et al., 2017; Li et al., 2017). Using these features obtained improved prediction results. In this work, we further improved prediction result by optimizing the extraction method of features. In the previous work, on the extraction of position conservation information of amino acids, some researchers used the position specific score matrix (PSSM) method to extract it (Sodhi et al., 2004; Hu et al., 2016a), whereas the other ones used the position weight scoring algorithm to extract its score values (Liu et al., 2019; Wang et al., 2019). However, the dimension of PSSM is excessively high, which will potentially lead to the overfitting problem, and the dimension of the score values is too low, which will lose a lot of information. Thus, we constructed the position weight matrices to extract the 2L-dimensional position conservation information of amino acids. In terms of extraction of the hydrophilic–hydrophobic information of amino acids, autocross covariance formula was attempted (Jiang et al., 2016). However, the method did not take into account the different number of amino acid species contained in each class of hydrophilic–hydrophobic properties. Same problem also exists in the classification of polarized charge. To settle these problems, we used the information entropy to extract the polarization charge and the hydrophilic–hydrophobic information of amino acids.

Dihedral angle of amino acid sequence can specify protein backbone conformation. Therefore, some researchers selected dihedral angle as feature and obtained improved results. But the extraction method, using two-dimensional real values of phi and psi angles as features (Hu et al., 2016b; Cui et al., 2019), ignored character of dihedral angle of each ion ligand–binding residue. In this work, the phi and psi angles were performed by using statistical analysis and reclassification, and they were extracted as feature parameters. The random forest (RF) algorithm is a strong classifier integrated with multiple weak classifiers. Fewer parameters are needed to be set, and it will cause less overfitting phenomenon in general. So, we finally used the RF algorithm to make the prediction model on the basis of combined feature parameters. By adding reclassified dihedral angle feature, we obtained improved prediction results and optimized the prediction model for each ion ligand–binding residue. Further, we compared our prediction results with the results of Artificial Neural Networks (ANNs) and Support Vector Machine (SVM) and turned out that the RF algorithm had a better prediction result.

## Materials and Methods

### Dataset

The dataset of ion ligand–binding residues constructed in this article was from the BioLip database. On the basis of literatures (Hu et al., 2016a; Cao et al., 2017), we selected the protein chains with the length longer than 50 residues and resolution below 3Å and then removed the protein chains whose pairwise sequence identity was higher than 30% by using the CD-HIT software (Li and Godzik, 2006). Inspired by the literature (Hu et al., 2016a), we finally selected the binding residues of 10 metal ion ligands (Zn^2+^, Cu^2+^, Fe^2+^, Fe^3+^, Co^2+^, Ca^2+^, Mg^2+^, Mn^2+^, Na^+^, K^+^) and four acid radical ion ligands (${\text{NO}}_{2}^{-}$, ${\text{CO}}_{3}^{2-}$, ${\text{SO}}_{4}^{2-}$, ${\text{PO}}_{4}^{3-}$) as research objects.

The interaction between the protein and ion ligand was related to both binding residues and their surrounding residues. Thus, we used the sliding window method to cut the protein chain into the corresponding sequence segments. If sequence segment center was an ion ligand–binding residue, it was defined as positive segment; otherwise, it was defined as the negative segment. In order to make each amino acid residue on the protein chains appear in the center of the sequence segment, we added (L-1)/2 virtual amino acids to the end of the protein chains. *L* is the sequence segment length. The non-redundant datasets of 14 ion ligands are shown in Table 1. By a number of calculations, the obtained optimal window lengths of Zn^2+^, Fe^2+^, Fe^3+^, Cu^2+^, Mn^2+^, Co^2+^, Ca^2+^, Mg^2+^, Na^+^, K^+^, ${\text{NO}}_{2}^{-}$, ${\text{CO}}_{3}^{2-}$, ${\text{SO}}_{4}^{2-}$, and ${\text{PO}}_{4}^{3-}$ were 7, 9, 9, 13, 7, 11, 9, 9, 9, 11, 13, 15, 13, and 13, respectively. The binding residues of 10 metal ion ligands and the corresponding optimal window lengths selected in this article were the same as in the literature (Cao et al., 2017).

**Table 1:** Benchmark datasets of 14 ion ligands.

**Ligands**		**Chains**	***P***	***N***
Metal ions	Zn^2+^	1428	6,408	405,113
	Cu^2+^	117	485	33,948
	Fe^2+^	92	382	29,345
	Fe^3+^	217	1,057	68,829
	Co^2+^	194	875	55,050
	Mn^2+^	459	2,124	156,625
	Ca^2+^	1237	6,789	396,957
	Mg^2+^	1461	5,212	480,307
	Na^+^	78	489	27,408
	K^+^	57	535	18,777
Acid radical ions	${\text{NO}}_{2}^{-}$	22	98	8,144
	${\text{CO}}_{3}^{2-}$	62	316	22,766
	${\text{SO}}_{4}^{2-}$	303	2,125	99,729
	${\text{PO}}_{4}^{3-}$	339	2,168	112,279

*P, is the number of binding residues; N, is the number of nonbinding residues*.

Because the number of negative segments was greater than positive segments, we used random sampling method in mathematics to balance the segments number in positive and negative datasets. Specifically, we randomly selected negative segments with the equal number of positive segments to compose negative datasets. In order to ensure the stability of the result, the negative samples were randomly sampled 10 times, and the final prediction result was the average value of the 10 results.

### The Selection and Extraction of Feature Parameters

#### Amino Acid Composition and Position Conservation Information

Our prestudy showed that amino acid composition of metal ion ligand–binding segments was different from amino acid composition of nonbinding segments (Cao et al., 2017). Besides, the literatures (Ansari and Raghava, 2010; Jiang et al., 2016; Li et al., 2017) also showed the amino acid composition was an important feature in the recognition work of ligand-binding residues. Therefore, the amino acid composition was selected as a feature parameter in this article.

According to the description in the literatures (Sodhi et al., 2004; Hu et al., 2016a,b; Cao et al., 2017; Li et al., 2017; Cui et al., 2019) and the statistical analysis of the position conservation in metal ion ligand–binding segments and nonbinding segments done by our group (Cao et al., 2017), we selected the amino acid position conservation information as a feature parameter. Because the dimension of PSSM (20^*^L) that is commonly used to extract position conservation information is excessively high, we constructed position weight matrices to extract position conservation information of amino acids (Kel et al., 2003; Gao and Hu, 2014). First, we constructed position weight matrices for positive and negative samples in the training set, respectively. The matrix element *m*_*i, j*_ of position weight matrix M was as follows:

(1) $$\begin{array}{l} m_{i,j}=\ln\;\left(\frac{p_{i,j}}{p_{0,j}}\right) \end{array}$$

(2) $$\begin{array}{l} p_{i,j}=\frac{\left(n_{i,j}+\frac{\sqrt{N_{i}}}{q}\right)}{\left(N_{i}+\sqrt{N_{i}}\right)} \end{array}$$

**Figure 1:**
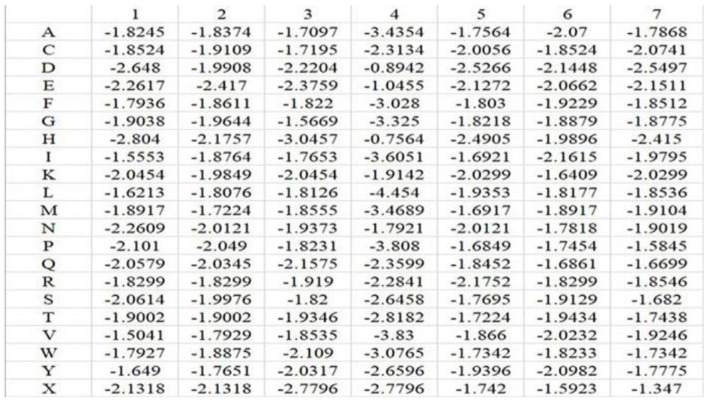
Position weight matrix constructed by the positive training samples of Mn^2+^ ligand.

**Figure 2:**
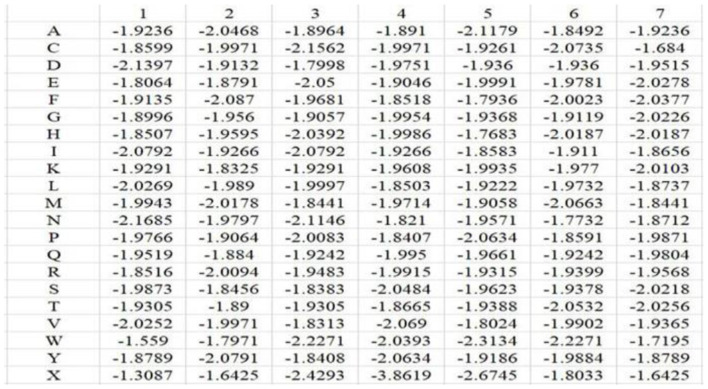
Position weight matrix constructed by the negative training samples of Mn^2+^ ligand.

In the above equation, *i* represents position, and *j* runs for 20 different amino acids and virtual residue “X”. *p*_*o, j*_ represents the background probability of the amino acid *j*, and *p*_*i, j*_ represents the probability of the amino acid *j* at the *i*^*th*^ position. *n*_*i, j*_ represents the frequency of amino acid *j* at the *i*^*th*^ position, and *N*_*i*_ is total number of amino acids at the *i*^*th*^ position. *q* represents the total number of categories, where *q* = 21. *L* is the length of the amino acid sequence segments. Taking Mn^2+^ ligand as an example, the position weight matrices constructed for its positive and negative training samples are shown in Figures 1, 2, respectively. For arbitrary amino acid sequence segment, such as “ACFPQSW,” according to the corresponding position weight matrices, we can get a 14-dimensional amino acid position conservation information: (−1.8245, −1.9236, −1.9109, −1.9971, −1.822, −1.9681, −3.808, −1.8407, −1.8452, −1.9661, −1.9129, −1.9378, −1.7342, −1.7195). Finally, the 2L-dimensional amino acid position conservation information was used as a feature parameter.

#### The Predicted Structural Information

It is well-known that structure of proteins directly determines their function, but not all proteins have experimentally measured 3D structure information. Because the secondary structure, RSA, and dihedral angle can reflect the local structure information of the protein, we selected above information as basic features (Hu et al., 2016a; Cao et al., 2017). In this article, the predicted secondary structure, RSA, and dihedral angle were obtained by ANGLOR software (Yang Zhang Lab; https://zhanglab.ccmb.med.umich.edu/ANGLOR/) (Wu and Zhang, 2008).

The secondary structure types that were predicted by the ANGLOR software included α-helix (H), β-strand (E), and coil (C). In previous studies, its composition and position conservation information were already used to predict ion ligand–binding residues and obtained great prediction results (Hu et al., 2016a,b; Cao et al., 2017; Li et al., 2017). Therefore, composition and 2L-dimensional position conservation features were extracted from secondary structure as feature parameters.

For the predicted RSA, its threshold value 0.25 was usually chosen to indicate whether the residue was exposed (RSA > 0.25) or buried (RSA < 0.25). In the literature (Cao et al., 2017), the four-classification of RSA was used as the basic feature parameter, and prediction results were obviously improved. Therefore, we selected four-classification of RSA in the literature (Cao et al., 2017) as a basic feature parameter and extracted its composition and 2L-dimensional position conservation information as predicted feature parameters. The four-classification of RSA in the literature (Cao et al., 2017) was as follows:

(3) $$\begin{array}{l} r\left(x\right)=\{\begin{array}{c} I,x\in \left(0,0.2\right]\\ J,x\in \left(0.2,0.45\right]\\ M,x\in \left(0.45,0.6\right]\\ N,x\in \left(0.6,0.85\right] \end{array} \end{array}$$

**Figure 3:**
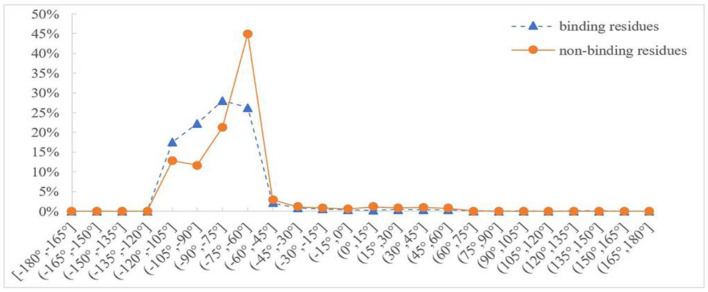
Distribution of phi angle for binding and nonbinding residues of Mn^2+^ ligand. The abscissa represents 24 classification intervals; the ordinate represents the percentage of predicted phi angle that appears in each interval.

**Figure 4:**
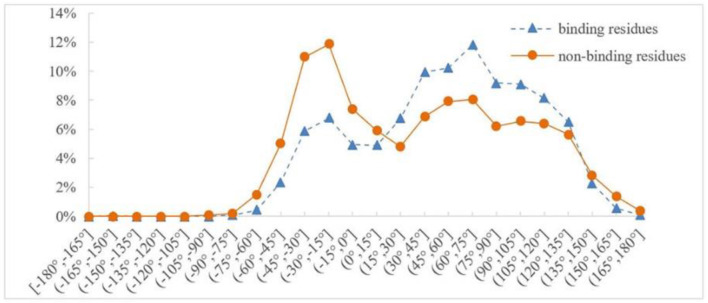
Distribution of psi angle for binding and nonbinding residues of Mn^2+^ ligand. The abscissa represents 24 classification intervals; the ordinate represents the percentage of predicted psi angle that appears in each interval.

Protein backbone dihedral angle specifies the backbone conformation of protein and is important for describing the local conformation of amino acids. Therefore, the dihedral angle information was selected as a basic feature parameter to predict the binding residues of the ion ligands. In a previous prediction work on binding sites of ligand–proteins, Chen et al. used 20 regions of the Ramachandran plot to calculate the value of propensity for ligand binding (Chen and Xu, 2016); Cui et al. used the real values of phi and psi angles to predict the ligand-binding residues on proteins (Cui et al., 2019). However, prediction results obtained by the above two methods did not achieve expectation. Therefore, the phi and psi angles of amino acid residues on protein chains binding with ion ligands were statistically analyzed and reclassified in this article. The degree of phi and psi angles predicted by ANGLOR software retained one decimal point, and the value range of phi and psi angles all was [−180°, 180°]. To simplify statistics, every 15th degree was divided into an interval; [−180°, 180°] was divided into 24 intervals. Then, we performed statistical analysis for the phi and psi angles. Taking Mn^2+^ ligand as an example, the distributions of phi and psi angles for binding and nonbinding residues are shown in Figures 3, 4, respectively.

In Figures 3, 4, the abscissa represents 24 classification intervals; the ordinate represents the percentage of predicted phi/psi angle appearing in each interval. We chose thresholds based on the difference of the predicted phi/psi angle between the binding and nonbinding residues. The threshold of phi angle was defined by the function *g(x)*, and the threshold of the psi angle was defined by the function *h(x)*. As can be seen from Figure 3, the difference of phi angle on the Mn^2+^ ligand-binding residues and nonbinding residues was mainly concentrated in two different intervals. Therefore, *g(x)* was defined as follows:

(4) $$\begin{array}{l} g\left(x\right)=\{\begin{array}{c} A,x\in \left[-180^{\circ },-75^{\circ }\right]\\ B,x\in \left[-75^{\circ },180^{\circ }\right] \end{array} \end{array}$$

Similarly, it can be seen from Figure 4 that the difference of psi angle on the Mn^2+^ ligand-binding residues and nonbinding residues was mainly concentrated in three intervals. Therefore, *h(x)* was defined as follows:

(5) $$\begin{array}{l} h\left(x\right)=\{\begin{array}{c} A,x\in \left[-180^{\circ },15^{\circ }\right]\\ B,x\in \left[15^{\circ },135^{\circ }\right]\\ C,x\in \left[135^{\circ },180^{\circ }\right] \end{array} \end{array}$$

In summary, the reclassification information of phi and psi angles was taken as the basic feature parameters, and its composition and the 2L-dimensional position conservation information were extracted as the feature parameters.

#### Physicochemical Properties of Amino Acids

**Figure 5:**
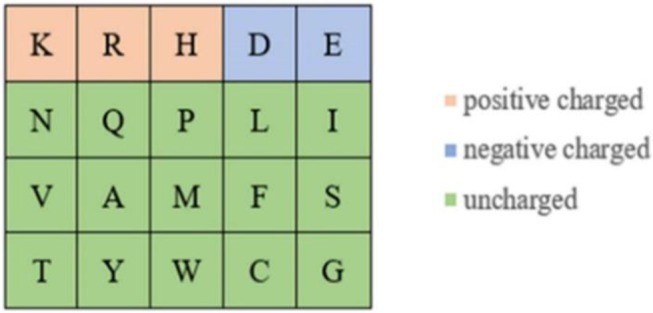
Polarization charge classifications of amino acids.

Because the ion ligand was charged, it was easy to bind to residues with opposite charge. Therefore, we selected the polarization charge information of amino acids as a basic feature parameter. The 20 amino acids were divided into three categories in this article (Taylor, 1986), as shown in Figure 5.

**Figure 6:**
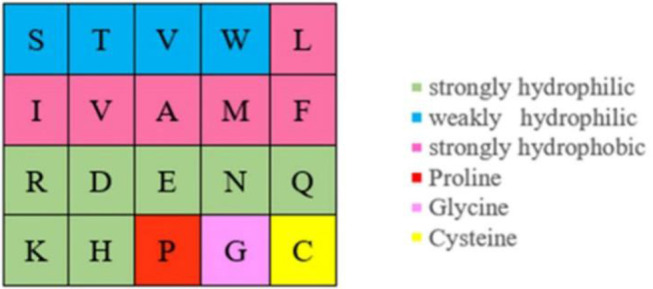
Hydrophilic–hydrophobic classifications of amino acids.

Actually, ion ligand mainly interacted with the amino acids exposed on the surface of the protein pocket. These amino acids generally showed hydrophilicity, so the hydrophilic–hydrophobic property of amino acids was also selected as a basic feature parameter. The 20 amino acids were divided into six categories according to hydrophilic–hydrophobic property (Pánek et al., 2005), as shown in Figure 6.

It can be seen from Figure 5 that the number of uncharged amino acids was many times more than charged amino acids, which made the information of charged amino acids unavailable. To solve this problem, information entropy was applied to extract the polarization charge information. Similarly, in the hydrophilic–hydrophobic classification of amino acids, *P, G*, and *C* were respectively divided into one category; the number of amino acids in these categories was far less than that in the other three categories. So, information entropy was also used on the extraction of hydrophilic–hydrophobic information.

Information entropy was proposed by Shannon (1948) to describe the uncertainty of information sources. Although the signal is uncertain, it can be measured according to the probability of its occurrence. For the state space *X*: {*x*_1_, *x*_2_, …, *x*_*q*_}, *n*_*j*_ is the occurrence number of *x*_*j*_ (*j* = 1, 2, …, *q*), and information entropy was defined as follows:

(6) $$\begin{array}{l} H\left(x\right)=-\overset{q}{\underset{j=1}{\sum }}p_{j}\log_{\text{2}}p_{j} \end{array}$$

(7) $$\begin{array}{l} p_{j}=\frac{\left(n_{j}+\frac{\sqrt{N}}{q}\right)}{\left(N+\sqrt{N}\right)} \end{array}$$

where, $N=\overset{q}{\underset{j=1}{\sum }}n_{j}.x_{j}$ represents hydrophilic–hydrophobic (or polarization charge) classifications of amino acids and virtual residue “X”. *p*_*j*_ represents the probability of the information symbol *x*_*j*_. When *x*_*j*_ represents hydrophilic–hydrophobic classifications of amino acids, *q* = 7, whereas when it represents polarization charge classifications of amino acids, *q* = 4. For arbitrary sequence segment, we can get the one-dimensional (1D) information entropy value according to Equation (6). Therefore, we used the 1D information entropy value as a feature.

### The RF Algorithm

The RF algorithm is a machine learning algorithm proposed by Breiman (2001). It is composed of multiple independent decision trees, each of which is a classifier. The basic principle is to integrate weak classifiers into one strong classifier, and the final result is determined by voting. The RF algorithm has been successfully applied to the prediction of β-hairpin and protein fold (Jia and Hu, 2011; Chen et al., 2014; Feng and Hu, 2014). The advantage of the RF algorithm is that fewer parameters need to be adjusted (Kandaswamy et al., 2010); in comparison with other algorithms, when the dimension is relatively high, the overfitting problem of RF algorithm is not so serious. The RF algorithm has two important parameters, one is the *m* ($m=\sqrt{M},$and *M* is the number of the feature initially selected), which is the size of a random feature subset for splitting the nodes; the other is the *k* that is the number of decision trees in the RF, generally *k* = 500 (Liaw and Wiener, 2002). In this article, we established our prediction model using the 4.6–12 RF algorithm package of R software version 3.4.3 (Vienna, Austria; https://www.R-project.org/).

### The Validation Methods and Evaluation Metrics

#### The Validation Methods

The constructed model was tested by the 5-fold cross-validation and independent test, which were commonly used on ligand–binding residues predictions (Sodhi et al., 2004; Lin et al., 2005; Hu et al., 2016a,b; Jiang et al., 2016; Cao et al., 2017; Li et al., 2017).

In the 5-fold cross-validation, the dataset was randomly divided into five subsets with same size. Each subset was regarded as test dataset in turn, and the rest of the four were accordingly as training dataset. After prediction process was repeated five times, the average value of five results was adopted as the final result.

In the independent test, the protein chains binding to each ion ligand were divided into two parts: one part accounted for 80% of the total protein chains, which was the training dataset for training the model; the other part accounted for 20% of the total protein chains, which was the independent test dataset for testing the model. Because the protein chains that generated the independent test dataset and the training dataset were different, the data of independent test dataset and training dataset were independent.

#### The Evaluation Metrics

The following four measures were used to evaluate the prediction performance of ion ligand–binding residues: sensitivity (Sn), specificity (Sp), accuracy (Acc), and Matthew's correlation coefficient (MCC) (Hu et al., 2016a,b; Lin et al., 2016; Cao et al., 2017; Li et al., 2017). These measures were defined as:

(8) $$\begin{array}{l} S_{n}=\frac{TP}{TP+FN}\times 100\% \end{array}$$

(9) $$\begin{array}{l} S_{p}=\frac{TN}{TN+FP}\times 100\% \end{array}$$

(10) $$\begin{array}{l} Acc=\frac{TP+TN}{TP+TN+FP+FN}\times 100\% \end{array}$$

(11) $$\begin{array}{l} MCC=\frac{\left(TP\times TN\right)-\left(FP\times FN\right)}{\sqrt{\left(TP\text{+}FP\right)\left(TP\text{+}FN\right)\left(TN\text{+}FP\right)\left(TN\text{+}FN\right)}} \end{array}$$

where TP represents the number of correctly predicted ion ligand–binding residues, TN represents the number of correctly predicted nonbinding residues, FP represents the number of nonbinding residues predicted as binding residues, and FN represents the number of binding residues predicted as nonbinding residues.

## Results and Discussion

### The Predicted Results of 5-Fold Cross-Validation

Because of the heavy imbalance in the original dataset, we took the number of positive segments as the standard and randomly sampled the equal number of negative segments 10 times, which generated 10 negative subsets. For each negative subset and the positive set, we extracted related feature parameters. Then, with five-fold cross-validation, we combined the feature parameters and inputted them into the RF algorithm. In this way, the progress was repeated 10 times, and the average result was taken as the result of one subset. Finally, we averaged the above results of 10 subsets as the prediction results.

#### The Prediction Results Obtained Without Adding Dihedral Angle Information

The composition and 2L-dimensional position conservation information of amino acids, secondary structure, RSA, and information entropy of polarization charge and hydrophilic–hydrophobic were used as feature parameters, and then we inputted them into the RF algorithm to predict the ion ligand–binding residues, and the predicted results obtained by the 5-fold cross-validation are shown in Table 2.

**Table 2:** Prediction results without adding dihedral angle information.

**Ligand**	***L***	**Sn (%)**	**Sp (%)**	**Acc (%)**	**MCC**
Zn^2+^	7	90.3	88.8	89.6	0.791
Cu^2+^	13	85.8	91.3	88.6	0.772
Fe^2+^	9	89.3	90.6	89.9	0.798
Fe^3+^	9	82.6	89.4	86.0	0.722
Co^2+^	11	76.8	86.2	81.5	0.632
Mn^2+^	7	77.8	86.3	82.1	0.644
Ca^2+^	9	71.7	76.5	74.1	0.483
Mg^2+^	9	71.6	82.2	76.9	0.541
Na^+^	9	71.8	71.8	71.8	0.436
K^+^	11	75.7	64.7	70.2	0.406
${\text{NO}}_{2}^{-}$	13	78.6	73.5	76.0	0.521
${\text{CO}}_{3}^{2-}$	15	72.2	76.9	74.5	0.491
${\text{SO}}_{4}^{2-}$	13	74.1	73.0	73.6	0.472
${\text{PO}}_{4}^{3-}$	13	76.1	78.2	77.1	0.543

*The above values were calculated on downsampling negative samples*.

**Figure 7:**
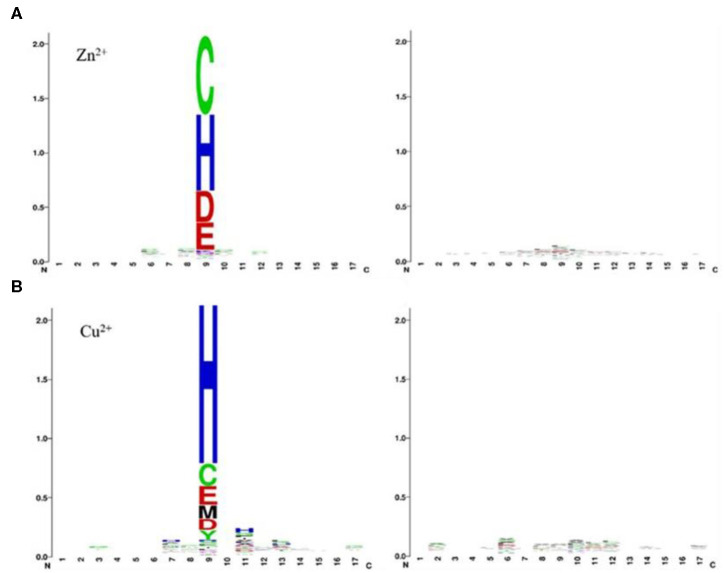
Position-specific conservation of amino acid residues in the binding and nonbinding sequence segments for Zn^2+^ **(A)**, Cu^2+^ **(B)**. Each ligand contains two subfigures, where the labeled subfigure is the position-specific conservation in binding residue sequence segments; the other is the position-specific conservation in nonbinding residue sequence segments [from literature (Cao et al., 2017)].

As can be seen from Table 2, comparing to the results of Ca^2+^, Mg^2+^, Na^+^, K^+^ ligands, the results of transition metal ions Zn^2+^, Cu^2+^, Fe^2+^, Fe^3+^, Co^2+^, and Mn^2+^ were better, with MCC value greater than 0.630, Acc value >81%, Sn value >76%, and Sp value >86%. This is because transition metal ion binding residues have a preference for conservation. For example, Zn^2+^ ligand–binding residues prefer to use C, H, D, E, and so on; Cu^2+^ ligand–binding residues prefer to use H, C, E, and so on (Cao et al., 2017). It was visualized in Figure 7.

#### The Prediction Results Obtained by Adding Dihedral Angle Information

**Figure 8:**
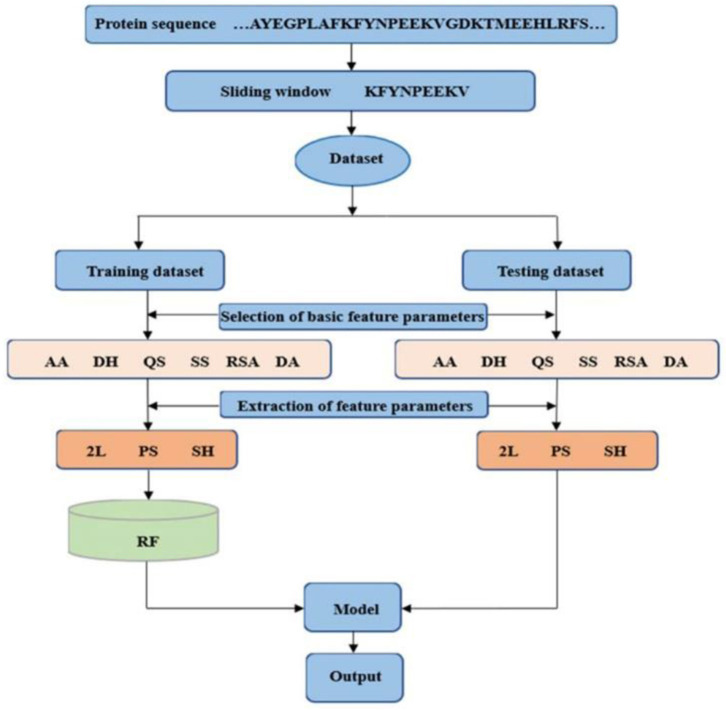
Flowchart of the proposed method. AA is amino acids; DH is polarization charge of amino acids; QS is hydrophilic and hydrophobic of amino acids; SS is predicted second structure; RSA is relative solvent accessibility; and DA is dihedral angle; 2L is 2L-dimensional position conservation feature; PS is composition feature; and SH is information entropy value.

The dihedral angle has an important influence on local structure of the protein backbone. The literatures (Chen and Xu, 2016; Hu et al., 2016a,b; Cui et al., 2019) showed that the dihedral angle played an important role in predicting the ligand binding sites on proteins. Therefore, composition feature and 2L-dimensional position conservation feature extracted from the phi and the psi angles were added to predict the binding residues of ion ligands. The flowchart of the proposed method was displayed in Figure 8. And the results obtained by the five-fold cross-validation are shown in Table 3.

**Table 3:** Prediction results with adding dihedral angle information by 5-fold cross-validation.

**Ligand**	***L***	**Sn (%)**	**Sp (%)**	**Acc (%)**	**MCC**
Zn^2+^	7	93.0 (99.8)	93.2 (99.5)	93.1 (99.7)	0.862 (0.993)
Cu^2+^	13	87.8 (95.5)	93.4 (97.1)	90.6 (96.3)	0.814 (0.926)
Fe^2+^	9	90.3 (91.9)	90.1 (90.7)	90.2 (91.3)	0.804 (0.826)
Fe^3+^	9	86.4 (86.9)	92.3 (88.7)	89.4 (87.8)	0.789 (0.756)
Co^2+^	11	86.1 (80.8)	88.2 (85.1)	87.1 (83.0)	0.743 (0.660)
Mn^2+^	7	84.9 (82.1)	89.6 (84.4)	87.3 (83.2)	0.747(0.664)
Ca^2+^	9	94.8 (71.3)	85.5 (79.1)	90.2 (74.8)	0.807 (0.502)
Mg^2+^	9	88.2 (76.6)	84.9 (73.9)	86.5 (75.3)	0.731 (0.505)
Na^+^	9	88.1 (82.2)	76.3 (76.2)	82.2 (79.4)	0.649 (0.586)
K^+^	11	89.3 (77.3)	71.0 (83.2)	80.2(80.3)	0.614 (0.607)
${\text{NO}}_{2}^{-}$	13	75.5	78.6	77.0	0.541
${\text{CO}}_{3}^{2-}$	15	80.7	82.0	81.3	0.627
${\text{SO}}_{4}^{2-}$	13	94.2	87.9	91.0	0.822
${\text{PO}}_{4}^{3-}$	13	91.5	90.9	91.4	0.827

*The above values were calculated on downsampling negative samples. The values in brackets are the 5-fold cross-validation results obtained by SVM in literature (Cao et al., 2017)*.

As shown in Table 3, prediction results were improved after adding dihedral angle information; the MCC values of Ca^2+^, Na^+^, K^+^, ${\text{SO}}_{4}^{2-}$, and ${\text{PO}}_{4}^{3-}$ ligands increased by at least 20 percentage points; the MCC values of Mn^2+^, Co^2+^, Mg^2+^, and ${\text{CO}}_{3}^{2-}$ increased by at least 10 percentage points; and the values of Acc, Sn, and Sp of these nine ion ligands were also significantly improved. The above values of other ion ligands were also slightly improved. The results showed that the ion ligand–binding residues were sensitive to the information of the reclassified dihedral angle. Namely, the information of the reclassified dihedral angle was effective for identifying ion ligand–binding residues.

#### The Comparison of the Predicted Results of 10 Metal Ion–Ligand Binding Residues

Because the datasets of metal ion ligand–binding residues used in this article and the optimal window length selected for each metal ion were the same as used in literature (Cao et al., 2017), the prediction results of 10 metal ion ligands obtained in this article were compared with prediction results obtained by SVM in literature (Cao et al., 2017).

In order to facilitate comparison, prediction results obtained by SVM were putted into brackets in Table 3. Apparently, the predicted results of Zn^2+^, Fe^2+^, and Cu^2+^ were lower than those obtained by SVM, but prediction results of the other seven metal ion ligands obtained by RF algorithm were all more accurate than those obtained by SVM. In particular, the MCC values of Ca^2+^ and Mg^2+^ were increased more than 20 percentage points comparing to SVM.

### The Prediction Results of Independent Test

In order to test the practicability of the model established in this article, independent test was performed. The independent test datasets of 14 ion ligands are shown in Table 4.

**Table 4:** Data of the training dataset and independent test dataset.

**Ligand**	**Training dataset**			**Independent test dataset**		
	**Chains**	***P***	***N***	**Chains**	***P***	***N***
Zn^2+^	1,142	5,145	321,161	286	1,263	83,952
Cu^2+^	93	377	27,548	24	108	6,400
Fe^2+^	73	301	23,824	19	81	5,521
Fe^3+^	173	859	54,945	44	198	13,884
Co^2+^	155	707	44,300	39	168	10,750
Mn^2+^	367	1,685	124,543	92	439	32,082
Ca^2+^	989	5,256	312,876	248	1,533	84,081
Mg^2+^	1,168	4,069	384,365	293	1,143	95,942
Na^+^	62	408	22,411	16	81	4,997
K^+^	45	410	14,882	12	125	3,895
${\text{NO}}_{2}^{-}$	17	76	6,218	5	22	1,926
${\text{CO}}_{3}^{2-}$	49	252	18,066	13	64	4,700
${\text{SO}}_{4}^{2-}$	242	1,751	79,164	61	374	20,565
${\text{PO}}_{4}^{3-}$	271	1,730	90,786	68	438	21,493

*P, is the number of binding residues; N, is the number of nonbinding residues*.

In the independent test, composition and 2L-dimensional position conservation information of the amino acids, secondary structure, RSA, and dihedral angle, as well as information entropy of polarization charge and hydrophilic–hydrophobic, were used as feature parameters and inputted into the RF algorithm to predict ion ligand–binding residues. The independent test results are shown in Table 5.

**Table 5:** Prediction results of the independent test.

**Ligand**	**Sn (%)**	**Sp (%)**	**Acc (%)**	**MCC**
Zn^2+^	92.2 (94.1)	90.7 (84.3)	90.7 (84.4)	0.326 (0.2528)
Cu^2+^	88.0 (91.7)	93.9 (82.9)	93.8 (83.0)	0.399 (0.2458)
Fe^2+^	79.0 (90.1)	93.7 (73.6)	93.5 (73.9)	0.333 (0.1708)
Fe^3+^	72.7 (87.9)	94.3 (72.7)	94.0 (72.9)	0.316 (0.1584)
Co^2+^	75.6 (73.2)	87.6 (82.3)	87.4 (82.2)	0.229 (0.1760)
Mn^2+^	72.9 (76.5)	91.9 (79.8)	91.7 (79.8)	0.262 (0.1599)
Ca^2+^	51.1 (59.5)	88.7 (79.2)	88.1 (78.9)	0.163 (0.1251)
Mg^2+^	74.6 (50.2)	81.8 (81.9)	81.7 (81.6)	0.150 (0.0871)
Na^+^	54.3 (33.3)	72.8 (78.2)	72.5 (77.5)	0.076 (0.0348)
K^+^	87.2 (45.6)	51.2 (62.8)	52.3 (62.3)	0.133 (0.0301)
${\text{NO}}_{2}^{-}$	86.4	95.4	95.3	0.377
${\text{CO}}_{3}^{2-}$	70.3	86.7	86.5	0.189
${\text{SO}}_{4}^{2-}$	50.8	88.4	87.7	0.158
${\text{PO}}_{4}^{3-}$	75.6	88.8	88.5	0.272

*The values in brackets are the independent test results obtained by SVM in literature (Cao et al., 2017)*.

The predicted results of Zn^2+^, Cu^2+^, Fe^2+^, Fe^3+^, Co^2+^, Mn^2+^, ${\text{NO}}_{2}^{-}$, and ${\text{PO}}_{4}^{3-}$ were improved, MCC values were higher than 0.220, the values of Acc and Sp were higher than 87%, and the Sn values were higher than 72%. The result of Cu^2+^ ligand was the highest, in which the MCC value was 0.399, and the values of Sn, Sp, and Acc were higher than 87%.The obtained results of Ca^2+^, Mg^2+^, Na^+^, K^+^, ${\text{CO}}_{3}^{2-}$, and ${\text{SO}}_{4}^{2-}$ ligands were lower, the MCC values were lower than 0.200, in which the predicted result of Na^+^ was the lowest, with the MCC value of 0.076 and the Acc value of 73.5%.

Because the independent test datasets of 10 metal ion ligands constructed in this article were the same as those used in the literature (Cao et al., 2017), the results obtained by independent test in this article were compared with those obtained by SVM in the literature (Cao et al., 2017). The results of the independent test in the literature (Cao et al., 2017) are shown in the brackets of Table 5.

Comparing MCC value, it is obvious that the independent test results obtained by the RF algorithm were better than those obtained by the SVM. In terms of values of the Acc and Sp, the RF algorithm achieved better predicted results of metal ion ligands, except for Na^+^ and K^+^ ligands. As for the Sn value, the prediction results of Co^2+^, Mg^2+^, Na^+^, and K^+^ ligands were better than those obtained by SVM, whereas other ligands were slightly lower. In general, the model constructed in this article was practical on the metal ion ligand–binding residues prediction.

### Comparison With SVM and ANN Algorithms

It is objective to compare our proposed methods with previous model using the same dataset. From Tables 3, 5, we found that most of the prediction results of this work were better than those of previous work (Cao et al., 2017).

In order to test the performance of our proposed method, we further made a comparison between RF and SVM and ANN algorithms on the same dataset, feature parameters, classification strategy, and evaluation methods. We inputted the same feature parameters extracted in this article into SVM and ANN algorithms to identify ion ligand–binding residues. The results obtained by the independent test are shown in Table 6. As seen, the prediction results of RF algorithm were best, although the results of Na^+^ and K^+^ ligands were slightly lower. At the same time, we can see that, when using the SVM on the same dataset, the prediction results obtained by selecting the feature parameters of this article were also better than those obtained in literature (Cao et al., 2017).

**Table 6:** Comparison of independent test results obtained by RF, SVM, and ANN.

**Ligands**	**Methods**	**Sn (%)**	**Sp (%)**	**Acc (%)**	**MCC**
Zn^2+^	RF	92.2	90.7	90.7	0.326
	SVM	93.4	88.6	88.7	0.298
	ANN	90.4	86.2	86.2	0.260
Cu^2+^	RF	88.0	93.9	93.8	0.399
	SVM	96.3	84.4	84.6	0.275
	ANN	87.0	93.0	92.9	0.371
Fe^2+^	RF	79.0	93.7	93.5	0.333
	SVM	96.3	80.1	80.3	0.224
	ANN	82.7	91.2	91.1	0.296
Fe^3+^	RF	72.7	94.3	94.0	0.316
	SVM	83.8	83.6	83.6	0.210
	ANN	78.8	87.2	87.0	0.225
Co^2+^	RF	75.6	87.6	87.4	0.229
	SVM	79.2	88.1	88.0	0.247
	ANN	77.4	84.2	84.1	0.203
Mn^2+^	RF	72.9	91.9	91.7	0.262
	SVM	79.3	86.5	86.4	0.217
	ANN	81.5	76.9	77.0	0.158
Ca^2+^	RF	51.1	88.7	88.1	0.163
	SVM	67.0	77.6	77.4	0.149
	ANN	69.2	65.5	65.6	0.097
Mg^2+^	RF	74.6	81.8	81.7	0.150
	SVM	77.2	78.9	78.8	0.141
	ANN	70.2	79.9	79.8	0.129
K^+^	RF	87.2	51.2	52.3	0.133
	SVM	80.0	70.1	70.4	0.187
	ANN	75.2	67.4	67.7	0.156
Na^+^	RF	54.3	72.8	72.5	0.076
	SVM	64.2	67.1	67.1	0.083
	ANN	44.4	80.4	79.8	0.078
${\text{NO}}_{2}^{-}$	RF	86.4	95.4	95.3	0.377
	SVM	59.1	96.2	95.8	0.284
	ANN	86.4	77.9	78.0	0.162
${\text{CO}}_{3}^{2-}$	RF	70.3	86.7	86.5	0.189
	SVM	76.6	83.9	83.8	0.186
	ANN	95.3	65.8	66.2	0.147
${\text{SO}}_{4}^{2-}$	RF	50.8	88.4	87.7	0.158
	SVM	83.4	62.2	62.6	0.124
	ANN	95.5	25.1	26.4	0.063
${\text{PO}}_{4}^{3-}$	RF	75.6	88.8	88.5	0.272
	SVM	88.1	72.0	72.4	0.185
	ANN	93.2	75.7	76.1	0.221

### The Optimization of Model

Because the reclassified phi and psi angles have a good effect on the prediction of ion ligand–binding residues, we further classified the phi and psi angles in the other way to optimize the prediction model for each ion ligand. At first, we classified the phi and psi angles by distribution. Therefore, we tried to classify the phi and the psi angles according to peak value. Taking Mn^2+^ ligand as an example, according to Figure 3, phi angle was divided into four categories and defined by function *f(x)*:

(12) $$\begin{array}{l} f\left(x\right)=\{\begin{array}{c} A,x\in \left[-180^{\circ },-1\text{0}5^{\circ }\right]\\ B,x\in \left(-105^{\circ }\text{,}-\text{7}{\text{5}}^{\circ }\right]\\ C,x\in \left(-\text{7}5^{\circ }\text{,}-\text{6}{\text{0}}^{\circ }\right]\\ D,x\in \left(-\text{6}{\text{0}}^{\circ },180^{\circ }\right] \end{array} \end{array}$$

According to Figure 4, psi angle was divided into three categories and defined by function *p(x)*:

(13) $$\begin{array}{l} p\left(x\right)=\{\begin{array}{c} A,x\in \left[-180^{\circ },-15^{\circ }\right]\\ B,x\in \left(-15^{\circ }\text{,7}{\text{5}}^{\circ }\right]\\ C,x\in \left(\text{7}5^{\circ }\text{,18}{\text{0}}^{\circ }\right] \end{array} \end{array}$$

**Figure 9:**
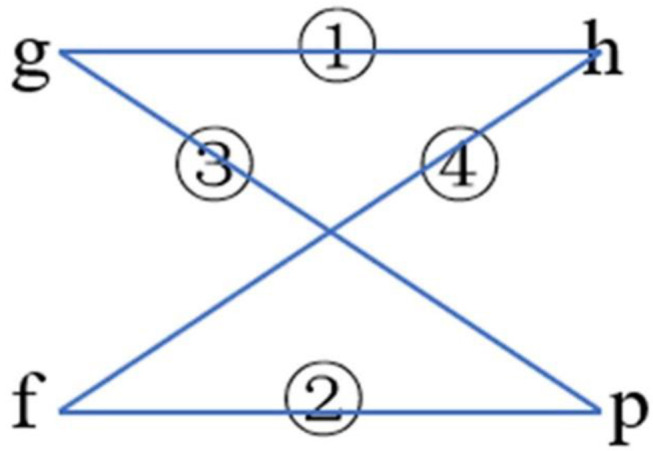
The combination ways of phi and psi angles.

The two classifications of phi angle and the two classifications of psi angle were combined, respectively (Figure 9). Their composition and 2L-dimensional position conservation information were extracted as the characteristic parameters.

The composition and 2L-dimensional position conservation information of amino acids, secondary structure, RSA, dihedral angle, and information entropy of polarization charge and hydrophilic–hydrophobic were used as characteristic parameters and inputted into the RF algorithm to predict ion ligand–binding residues. The optimal prediction model and the corresponding prediction result of each ion ligand obtained by the five-fold cross-validation are shown in Table 7.

**Table 7:** Optimal Predicted models of ion ligands and corresponding predicted results.

**Ligand**	**Model**	**Sn (%)**	**Sp (%)**	**Acc (%)**	**MCC**
Zn^2+^	➂	92.9	93.5	93.2	0.865
Cu^2+^	➀	87.8	93.4	90.6	0.814
Fe^2+^	➀	90.3	90.1	90.2	0.804
Fe^3+^	➂	87.3	92.1	89.7	0.796
Co^2+^	➃	87.2	91.0	89.1	0.782
Mn^2+^	➁	86.3	90.9	88.6	0.773
Ca^2+^	➀	94.8	85.5	90.2	0.807
Mg^2+^	➂	88.5	86.3	87.4	0.749
K^+^	➃	90.3	75.9	83.1	0.669
Na^+^	➀	88.1	76.3	82.2	0.649
${\text{NO}}_{2}^{-}$	➀	75.5	78.6	77.0	0.541
${\text{CO}}_{3}^{2-}$	➂	81.3	84.8	83.1	0.662
${\text{SO}}_{4}^{2-}$	➂	94.0	90.3	92.1	0.843
${\text{PO}}_{4}^{3-}$	➂	92.3	92.5	92.4	0.849

As seen, not all ion ligand–binding residues were sensitive to the first combination way of phi and psi angles. For example, the optimal prediction model of Mn^2+^ ligand corresponded to the second combination way of phi and psi angles, and the optimal prediction model of Zn^2+^ ligand corresponded to the third combination way of phi and psi angles. It can be seen that the different reclassification and combination ways of phi and psi angles have an important impact on the prediction of ion ligand–binding residues. Therefore, when using the reclassified dihedral angle information to predict ion ligand–binding residues, different classifications and combination ways of dihedral angle (phi and psi angles) should be considered to optimize the prediction model of binding residues of each ion ligand.

## Conclusion

Many proteins perform their functions by interacting with ion ligands. To illustrate the protein functions, it is a significant work to recognize the ion ligand–binding residues. In this article, based on optimized dihedral angle, we predicted the 14 ion ligand–binding residues from the BioLip database by RF algorithm and obtained improved results. During the progress, the dihedral angle information was statistically analyzed and reclassified. Besides, the new extraction methods of feature parameter were proposed, in which the position weight matrices were constructed to extract the 2L-dimensional position conservation features; the polarization charge information and hydrophilic–hydrophobic information of amino acids were extracted by using information entropy. These changes in extraction methods improved the predicted results of ion ligand–binding residues. In particular, the reclassification information of the dihedral angle has significantly improved the prediction results of the ion ligand–binding residues, indicating that the reclassification information of the dihedral angle is an important feature parameter for the identification of the ion ligand–binding residues. By classifying and combining phi and psi angles, we optimized the prediction model for each ion ligand–binding residue. Thus, with different classification standards and combined methods of the dihedral angle (phi and psi angles), it can further improve the prediction results of ion ligand–binding residues.

## Data Availability Statement

The datasets used and analyzed during the current study are available from the corresponding author on reasonable request.

## Author Contributions

LL performed the experiments and wrote the paper. XH designed the experiments and analyzed the results. ZF, SW, SX, and KS gave guidance on the writing of the paper. All authors read and approved the final manuscript.

## Conflict of Interest

The authors declare that the research was conducted in the absence of any commercial or financial relationships that could be construed as a potential conflict of interest.
